# *N*-(1,3-Dioxo-2,3-dihydro-1*H*-isoindol-2-yl)-4,4′′-difluoro-5′-hy­droxy-1,1′:3′,1′′-terphenyl-4′-carboxamide

**DOI:** 10.1107/S160053681203365X

**Published:** 2012-08-01

**Authors:** Hoong-Kun Fun, Tze Shyang Chia, S. Samshuddin, B. Narayana, B. K. Sarojini

**Affiliations:** a X-ray Crystallography Unit, School of Physics, Universiti Sains Malaysia, 11800 USM, Penang, Malaysia; b Department of Studies in Chemistry, Mangalore University, Mangalagangotri 574 199, India; c Department of Chemistry, P. A. College of Engineering, Nadupadavu, Montepadavu, PO, Mangalore 574 153, India

## Abstract

The asymmetric unit of the title compound, C_27_H_16_F_2_N_2_O_4_, consists of two crystallographically independent mol­ecules (*A* and *B*). In mol­ecule *B*, the isoindoline-1,3-dione ring system is disordered over two set of sites with a site-occupancy ratio of 0.658 (12):0.342 (12). In mol­ecule *A*, the fluoro-substituted benzene rings make dihedral angles of 18.36 (8) and 46.37 (8)° with the central benzene ring, whereas the corresponding angles are 40.90 (8) and 52.89 (9)° in mol­ecule *B*. The isoindoline ring system in mol­ecule *A* and the major and minor components of the disordered isoindoline ring system in mol­ecule *B* make dihedral angles of 58.50 (4), 54.13 (16) and 70.01 (28) °, respectively, with their attached benzene rings, linked through the amide group. An intra­molecular O—H⋯O hydrogen bond generates an *S*(6) ring in each mol­ecule. In the crystal, mol­ecules are linked by N—H⋯O, C—H⋯F and C—H⋯O hydrogen bonds into sheets lying parallel to the *bc* plane. The crystal studied was a non-merohedral twin with a refined twin component ratio of 0.9316 (8):0.0684 (8).

## Related literature

For related structures and background to terphenyls and their oxadiazole derivatives, see: Fun *et al.* (2012*a* ▶,*b* ▶); Samshuddin *et al.* (2011) ▶. For the planarity of isoindoline, see: Asad *et al.* (2011 ▶). For hydrogen-bond motifs, see: Bernstein *et al.* (1995 ▶). For the stability of the temperature controller used in the data collection, see: Cosier & Glazer (1986 ▶).
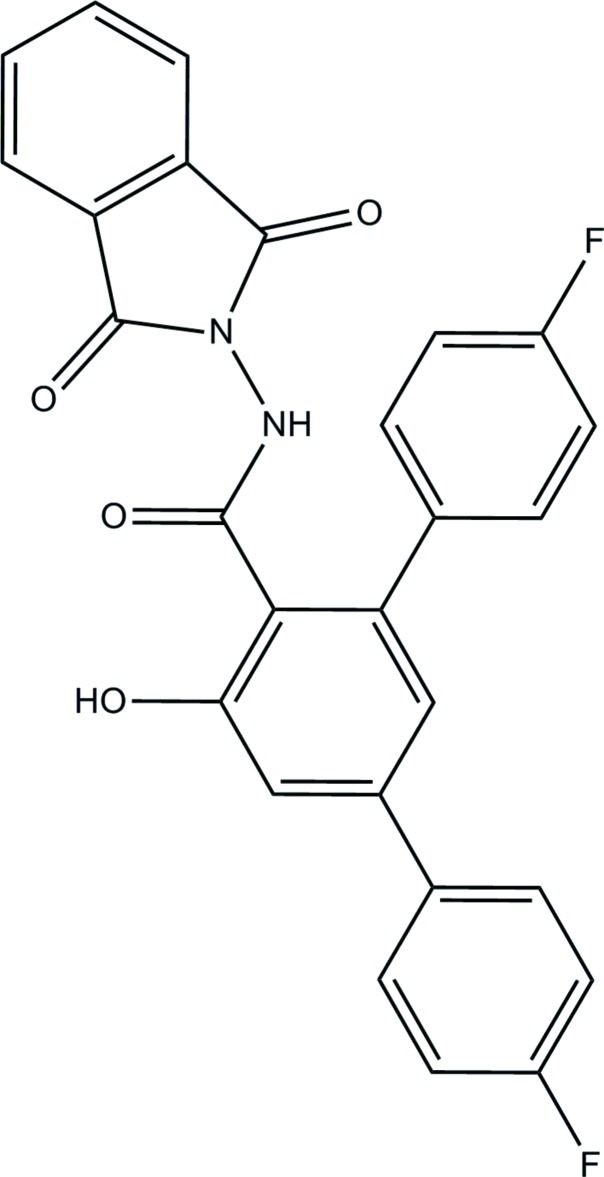


## Experimental

### 

#### Crystal data

C_27_H_16_F_2_N_2_O_4_
*M* *_r_* = 470.42
Monoclinic, 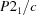

*a* = 24.8732 (10) Å
*b* = 8.9875 (4) Å
*c* = 21.3722 (9) Å
β = 114.921 (1)°
*V* = 4332.9 (3) Å^3^
*Z* = 8
Mo *K*α radiation
μ = 0.11 mm^−1^
*T* = 100 K
0.41 × 0.31 × 0.14 mm

#### Data collection

Bruker APEX DUO CCD diffractometer
Absorption correction: multi-scan (*SADABS*; Bruker, 2009 ▶) *T*_min_ = 0.956, *T*_max_ = 0.985
16713 measured reflections
16713 independent reflections
12698 reflections with *I* > 2σ(*I*)

#### Refinement

*R*[*F* ^2^ > 2σ(*F* ^2^)] = 0.056
*wR*(*F* ^2^) = 0.168
*S* = 1.05
16713 reflections
748 parameters
45 restraints
H atoms treated by a mixture of independent and constrained refinement
Δρ_max_ = 0.63 e Å^−3^
Δρ_min_ = −0.90 e Å^−3^

### 

Data collection: *APEX2* (Bruker, 2009 ▶); cell refinement: *SAINT* (Bruker, 2009 ▶); data reduction: *SAINT*; program(s) used to solve structure: *SHELXTL* (Sheldrick, 2008 ▶); program(s) used to refine structure: *SHELXTL*; molecular graphics: *SHELXTL*; software used to prepare material for publication: *SHELXTL* and *PLATON* (Spek, 2009 ▶).

## Supplementary Material

Crystal structure: contains datablock(s) global, I. DOI: 10.1107/S160053681203365X/hb6906sup1.cif

Structure factors: contains datablock(s) I. DOI: 10.1107/S160053681203365X/hb6906Isup2.hkl

Supplementary material file. DOI: 10.1107/S160053681203365X/hb6906Isup3.cml

Additional supplementary materials:  crystallographic information; 3D view; checkCIF report

## Figures and Tables

**Table 1:** Hydrogen-bond geometry (Å, °)

*D*—H⋯*A*	*D*—H	H⋯*A*	*D*⋯*A*	*D*—H⋯*A*
N1*A*—H1*NA*⋯O2*A* ^i^	0.80 (3)	2.02 (3)	2.8000 (19)	166 (3)
O1*B*—H1*OB*⋯O2*B*	0.88 (3)	1.79 (4)	2.5663 (17)	145 (4)
N1*B*—H1*NB*⋯O2*B* ^ii^	0.91 (3)	2.04 (3)	2.779 (2)	138 (2)
O1*A*—H1*OA*⋯O2*A*	0.82 (3)	1.94 (3)	2.6402 (18)	143 (3)
C2*A*—H2*AA*⋯F2*A* ^iii^	0.93	2.45	3.160 (2)	133
C4*B*—H4*BA*⋯O3*B* ^iv^	0.93	2.41	3.271 (7)	154
C14*B*—H14*B*⋯O3*B* ^v^	0.93	2.44	3.293 (6)	152
C25*B*—H25*B*⋯O1*B* ^vi^	0.93	2.47	3.209 (3)	136

Symmetry codes: (i) 
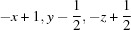
; (ii) 
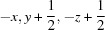
; (iii) 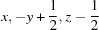; (iv) 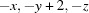; (v) 
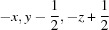
; (vi) 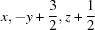.

**Fig. 1.**
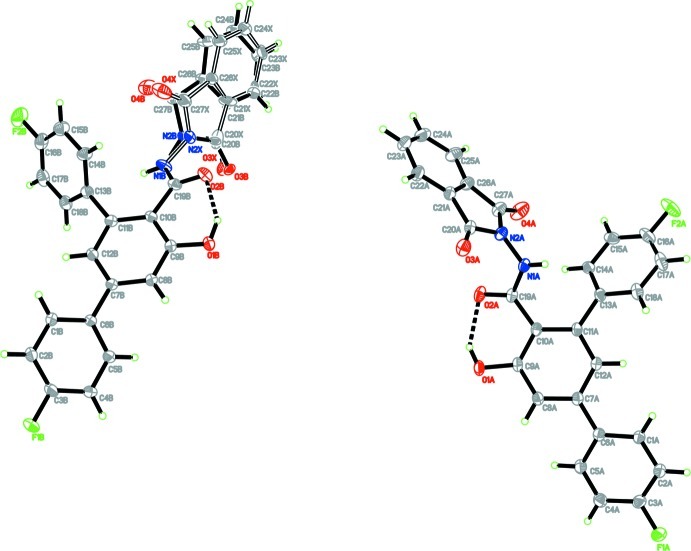
The molecular structure of the title compound with 50% probability displacement ellipsoids. Intramolecular hydrogen bonds are shown by dashed lines.

**Fig. 2.**
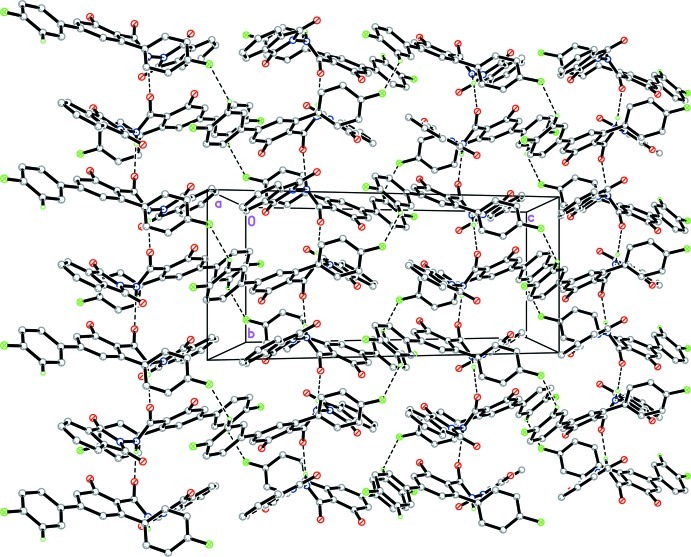
The crystal packing of the title compound. The dashed lines represent the hydrogen bonds.

## supplementary crystallographic information

### Comment

In continuation of our work on synthesis of various terphenyl derivatives (Fun *et al.*, 2012*a*,*b*), the title compound was prepared and its crystal structure is reported. The starting material of the title compound was prepared from 4,4'-difluoro chalcone by several steps (Samshuddin *et al.*, 2011).

The asymmetric unit of the title compound consists of two crystallographically independent molecules (*A* and *B*) as shown in Fig. 1. Each molecule contains one benzene ring [C7–C12], two fluoro-substituted benzene rings [C1–C6 & C13–C18] and an isoindoline-1,3-dione ring system [N2/O3/O4/C20–C27]. In molecule *B*, isoindoline-1,3-dione ring system is disordered over two positions with a site-occupancy ratio of 0.658 (12):0.342 (12). In molecule *A*, the fluoro-substituted benzene rings make dihedral angles of 18.36 (8) and 46.37 (8)° with the C7–C12 benzene ring, whereas the corresponding angles are 40.90 (8) and 52.89 (9)° in molecule *B*. The isoindoline ring systems [N2A/C20A–C27A, N2B/C20B–C27B & N2X/C20X–C27X; maximum deviations = 0.035 (1), 0.075 (4) and 0.084 (18) Å, respectively] make dihedral angles of 58.50 (4), 54.13 (16) and 70.01 (28) °, respectively with their attached C7–C12 benzene ring. An intramolecular O—H···O hydrogen bond (Table 1) generates an S(6) ring motif (Fig. 1; Bernstein *et al.*, 1995) in each molecule. The bond lengths and angles are comparable to those found in related structures (Fun *et al.*, 2012*a*,*b*)

In the crystal (Fig. 2), molecules are linked by N1A—H1NA···O2A, N1B—H1NB···O2B, C2A—H2AA···F2A, C4B—H4BA···O3B, C14B—H14B···O3B and C25B—H25B···O1B hydrogen bonds into sheets parallel to *bc* plane.

### Experimental

A mixture of 4,4''-difluoro-5'-hydroxy-1,1':3',1''-terphenyl-4'-carbohydrazide (3.40 g, 0.01 mol) and phthalic anhydride (1.48 g, 0.01 mol) was dissolved in acetic acid (25 ml) and heated to reflux for 6 h. The reaction mixture was then poured into ice cold water, filtered and crystallized from ethanol. Colourless blocks were grown from DMF solution by slow evaporation method and yield of the compound was 76%. (m.p. 506 K).

### Refinement

N-bound and O-bound H atoms were located in a difference fourier map and refined freely [N1A—H1NA = 0.80 (3) Å, N1B—H1NB = 0.91 (3) Å, O1A—H1OA = 0.82 (3) Å, O1B—H1OB = 0.88 (3) Å]. The remaining H atoms were positioned geometrically [C—H = 0.93 Å] and refined using a riding model with *U*_iso_(H) = 1.2*U*_eq_(C). FLAT restraint was applied to the N2/O3/O4/C20–C27 ring system in molecule *A* and minor component of molecule *B* so that the planarity of isoindoline ring is agreed with that found in a related structure (Asad *et al.*, 2011). Ten outliers, (0 2 3), (1 0 0), (3 3 5), (20 0 6), (12 0 6), (21 3 6), (10 1 7), (15 6 6), (7 4 6) and (14 5 6), were omitted in the final refinement. The crystal studied was a non-merohedral twin with BASF = 0.0684 (8).

### Crystal data

**Table:**

C_27_H_16_F_2_N_2_O_4_	*F*(000) = 1936
*M**_r_* = 470.42	*D*_x_ = 1.442 Mg m^−^^3^
Monoclinic, *P*2_1_/*c*	Mo *K*α radiation, λ = 0.71073 Å
Hall symbol: -P 2ybc	Cell parameters from 9881 reflections
*a* = 24.8732 (10) Å	θ = 2.8–33.2°
*b* = 8.9875 (4) Å	µ = 0.11 mm^−^^1^
*c* = 21.3722 (9) Å	*T* = 100 K
β = 114.921 (1)°	Block, colourless
*V* = 4332.9 (3) Å^3^	0.41 × 0.31 × 0.14 mm
*Z* = 8	

### Data collection

**Table:**

Bruker APEX DUO CCD diffractometer	16713 independent reflections
Radiation source: fine-focus sealed tube	12698 reflections with *I* > 2σ(*I*)
Graphite monochromator	*R*_int_ = 0.000
φ and ω scans	θ_max_ = 33.3°, θ_min_ = 0.9°
Absorption correction: multi-scan (*SADABS*; Bruker, 2009)	*h* = −38→34
*T*_min_ = 0.956, *T*_max_ = 0.985	*k* = −13→13
16713 measured reflections	*l* = 0→32

### Refinement

**Table:**

Refinement on *F*^2^	Primary atom site location: structure-invariant direct methods
Least-squares matrix: full	Secondary atom site location: difference Fourier map
*R*[*F*^2^ > 2σ(*F*^2^)] = 0.056	Hydrogen site location: inferred from neighbouring sites
*wR*(*F*^2^) = 0.168	H atoms treated by a mixture of independent and constrained refinement
*S* = 1.05	*w* = 1/[σ^2^(*F*_o_^2^) + (0.0737*P*)^2^ + 2.8746*P*] where *P* = (*F*_o_^2^ + 2*F*_c_^2^)/3
16713 reflections	(Δ/σ)_max_ < 0.001
748 parameters	Δρ_max_ = 0.63 e Å^−^^3^
45 restraints	Δρ_min_ = −0.90 e Å^−^^3^

### Special details

**Table:**

Experimental. The crystal was placed in the cold stream of an Oxford Cryosystems Cobra open-flow nitrogen cryostat (Cosier & Glazer, 1986) operating at 100.0 (1) K.
Geometry. All e.s.d.'s (except the e.s.d. in the dihedral angle between two l.s. planes) are estimated using the full covariance matrix. The cell e.s.d.'s are taken into account individually in the estimation of e.s.d.'s in distances, angles and torsion angles; correlations between e.s.d.'s in cell parameters are only used when they are defined by crystal symmetry. An approximate (isotropic) treatment of cell e.s.d.'s is used for estimating e.s.d.'s involving l.s. planes.
Refinement. Refinement of *F*^2^ against ALL reflections. The weighted *R*-factor *wR* and goodness of fit *S* are based on *F*^2^, conventional *R*-factors *R* are based on *F*, with *F* set to zero for negative *F*^2^. The threshold expression of *F*^2^ > σ(*F*^2^) is used only for calculating *R*-factors(gt) *etc*. and is not relevant to the choice of reflections for refinement. *R*-factors based on *F*^2^ are statistically about twice as large as those based on *F*, and *R*- factors based on ALL data will be even larger.

### Fractional atomic coordinates and isotropic or equivalent isotropic displacement parameters (Å^2^)

**Table:**

	*x*	*y*	*z*	*U*_iso_*/*U*_eq_	Occ. (<1)
F1A	0.70185 (5)	0.57672 (16)	−0.07644 (6)	0.0364 (3)	
F2A	0.74047 (5)	0.31572 (16)	0.47234 (7)	0.0448 (3)	
O1A	0.44381 (5)	0.67527 (14)	0.09291 (6)	0.0239 (2)	
O2A	0.45373 (5)	0.64374 (13)	0.22015 (7)	0.0244 (2)	
O3A	0.37372 (5)	0.32205 (15)	0.22722 (7)	0.0272 (2)	
O4A	0.52736 (6)	0.57608 (18)	0.39267 (8)	0.0384 (3)	
N1A	0.49213 (6)	0.42265 (15)	0.26521 (7)	0.0194 (2)	
N2A	0.46069 (6)	0.43104 (14)	0.30464 (7)	0.0200 (2)	
C1A	0.66264 (7)	0.46269 (19)	0.06103 (8)	0.0220 (3)	
H1AA	0.6752	0.3957	0.0976	0.026*	
C2A	0.69280 (7)	0.4694 (2)	0.01934 (9)	0.0255 (3)	
H2AA	0.7255	0.4091	0.0278	0.031*	
C3A	0.67274 (7)	0.5687 (2)	−0.03518 (9)	0.0258 (3)	
C4A	0.62450 (7)	0.6598 (2)	−0.04955 (9)	0.0262 (3)	
H4AA	0.6116	0.7240	−0.0872	0.031*	
C5A	0.59568 (7)	0.65329 (19)	−0.00633 (9)	0.0233 (3)	
H5AA	0.5637	0.7160	−0.0146	0.028*	
C6A	0.61381 (7)	0.55424 (17)	0.04938 (8)	0.0193 (3)	
C7A	0.58244 (6)	0.54752 (17)	0.09474 (8)	0.0185 (2)	
C8A	0.52569 (6)	0.60577 (17)	0.07381 (8)	0.0194 (3)	
H8AA	0.5062	0.6442	0.0295	0.023*	
C9A	0.49761 (6)	0.60762 (17)	0.11803 (8)	0.0187 (3)	
C10A	0.52419 (6)	0.54063 (16)	0.18387 (8)	0.0177 (2)	
C11A	0.58238 (6)	0.48200 (16)	0.20595 (8)	0.0177 (2)	
C12A	0.61013 (6)	0.48774 (17)	0.16141 (8)	0.0186 (3)	
H12A	0.6484	0.4506	0.1764	0.022*	
C13A	0.61965 (6)	0.42961 (17)	0.27708 (8)	0.0190 (3)	
C14A	0.62408 (7)	0.51251 (19)	0.33414 (8)	0.0215 (3)	
H14A	0.5999	0.5952	0.3279	0.026*	
C15A	0.66434 (7)	0.4728 (2)	0.40044 (9)	0.0270 (3)	
H15A	0.6673	0.5278	0.4386	0.032*	
C16A	0.69952 (8)	0.3503 (2)	0.40793 (10)	0.0319 (4)	
C17A	0.69626 (8)	0.2637 (2)	0.35338 (11)	0.0319 (4)	
H17A	0.7204	0.1806	0.3604	0.038*	
C18A	0.65589 (7)	0.30408 (19)	0.28748 (10)	0.0254 (3)	
H18A	0.6529	0.2471	0.2498	0.030*	
C19A	0.48859 (6)	0.54052 (16)	0.22477 (8)	0.0188 (3)	
C20A	0.40094 (6)	0.38583 (17)	0.28064 (8)	0.0192 (3)	
C21A	0.38250 (7)	0.43651 (17)	0.33479 (8)	0.0199 (3)	
C22A	0.32915 (7)	0.4177 (2)	0.33958 (9)	0.0266 (3)	
H22A	0.2982	0.3641	0.3066	0.032*	
C23A	0.32378 (8)	0.4828 (2)	0.39620 (10)	0.0297 (3)	
H23A	0.2885	0.4722	0.4009	0.036*	
C24A	0.36994 (9)	0.5627 (2)	0.44533 (10)	0.0308 (4)	
H24A	0.3649	0.6058	0.4820	0.037*	
C25A	0.42400 (9)	0.5795 (2)	0.44060 (10)	0.0305 (4)	
H25A	0.4553	0.6315	0.4739	0.037*	
C26A	0.42891 (7)	0.51557 (19)	0.38443 (9)	0.0228 (3)	
C27A	0.47978 (7)	0.51708 (19)	0.36511 (9)	0.0243 (3)	
F1B	−0.24301 (5)	0.91133 (16)	−0.29187 (5)	0.0347 (3)	
F2B	−0.22044 (6)	1.19023 (17)	0.26237 (7)	0.0440 (3)	
O1B	0.02966 (5)	0.75887 (17)	0.11463 (6)	0.0279 (3)	
O2B	0.03502 (6)	0.78615 (14)	0.23665 (6)	0.0256 (2)	
N1B	−0.00874 (7)	0.98785 (17)	0.25626 (7)	0.0249 (3)	
C1B	−0.20753 (7)	0.87878 (19)	−0.11017 (8)	0.0210 (3)	
H1BA	−0.2263	0.8584	−0.0816	0.025*	
C2B	−0.24027 (7)	0.8785 (2)	−0.18146 (8)	0.0239 (3)	
H2BA	−0.2806	0.8575	−0.2010	0.029*	
C3B	−0.21122 (7)	0.9102 (2)	−0.22228 (8)	0.0242 (3)	
C4B	−0.15137 (7)	0.9400 (2)	−0.19595 (8)	0.0270 (3)	
H4BA	−0.1331	0.9604	−0.2250	0.032*	
C5B	−0.11913 (7)	0.9385 (2)	−0.12468 (8)	0.0245 (3)	
H5BA	−0.0786	0.9572	−0.1058	0.029*	
C6B	−0.14672 (6)	0.90931 (18)	−0.08102 (7)	0.0189 (3)	
C7B	−0.11148 (6)	0.90581 (18)	−0.00535 (7)	0.0192 (3)	
C8B	−0.05588 (7)	0.8402 (2)	0.02207 (8)	0.0222 (3)	
H8BA	−0.0403	0.8024	−0.0073	0.027*	
C9B	−0.02312 (6)	0.83030 (19)	0.09311 (8)	0.0204 (3)	
C10B	−0.04459 (7)	0.89221 (17)	0.13910 (7)	0.0180 (2)	
C11B	−0.10190 (7)	0.95784 (17)	0.11118 (7)	0.0184 (2)	
C12B	−0.13399 (7)	0.96299 (18)	0.03979 (8)	0.0196 (3)	
H12B	−0.1715	1.0057	0.0215	0.023*	
C13B	−0.13197 (7)	1.01929 (18)	0.15303 (8)	0.0204 (3)	
C14B	−0.13921 (8)	0.9340 (2)	0.20379 (9)	0.0255 (3)	
H14B	−0.1241	0.8378	0.2128	0.031*	
C15B	−0.16887 (9)	0.9922 (2)	0.24094 (10)	0.0309 (4)	
H15B	−0.1736	0.9359	0.2748	0.037*	
C16B	−0.19113 (8)	1.1348 (2)	0.22641 (10)	0.0306 (4)	
C17B	−0.18557 (8)	1.2222 (2)	0.17653 (9)	0.0289 (3)	
H17B	−0.2012	1.3179	0.1677	0.035*	
C18B	−0.15578 (7)	1.16279 (19)	0.13971 (9)	0.0237 (3)	
H18B	−0.1517	1.2197	0.1056	0.028*	
C19B	−0.00406 (7)	0.88355 (17)	0.21341 (8)	0.0202 (3)	
O3B	0.1114 (3)	1.0874 (6)	0.3230 (3)	0.0205 (7)	0.658 (12)
O4B	−0.03851 (17)	0.8627 (7)	0.36124 (16)	0.0433 (10)	0.658 (12)
N2B	0.0251 (2)	0.9875 (5)	0.3257 (2)	0.0126 (6)	0.658 (12)
C20B	0.0845 (3)	1.0357 (9)	0.3540 (2)	0.0160 (14)	0.658 (12)
C21B	0.1069 (2)	1.0054 (8)	0.4280 (3)	0.0175 (13)	0.658 (12)
C22B	0.1588 (4)	1.0456 (16)	0.4820 (4)	0.0235 (17)	0.658 (12)
H22B	0.1884	1.0945	0.4742	0.028*	0.658 (12)
C23B	0.1665 (3)	1.0115 (9)	0.5491 (4)	0.0254 (13)	0.658 (12)
H23B	0.2018	1.0369	0.5862	0.031*	0.658 (12)
C24B	0.1228 (2)	0.9414 (8)	0.56112 (18)	0.0370 (11)	0.658 (12)
H24B	0.1293	0.9180	0.6061	0.044*	0.658 (12)
C25B	0.0686 (2)	0.9044 (8)	0.50666 (16)	0.0413 (14)	0.658 (12)
H25B	0.0388	0.8569	0.5146	0.050*	0.658 (12)
C26B	0.0612 (2)	0.9412 (6)	0.44076 (18)	0.0250 (8)	0.658 (12)
C27B	0.00860 (19)	0.9190 (6)	0.37426 (18)	0.0258 (8)	0.658 (12)
O3X	0.1052 (6)	1.1164 (11)	0.3266 (7)	0.0297 (19)	0.342 (12)
O4X	−0.0235 (4)	0.7929 (13)	0.3521 (4)	0.046 (3)	0.342 (12)
N2X	0.0342 (4)	0.9593 (9)	0.3252 (5)	0.0195 (16)	0.342 (12)
C20X	0.0880 (7)	1.0363 (19)	0.3594 (6)	0.029 (4)	0.342 (12)
C21X	0.1074 (6)	1.0017 (19)	0.4334 (6)	0.031 (4)	0.342 (12)
C22X	0.1601 (7)	1.041 (2)	0.4886 (8)	0.022 (3)	0.342 (12)
H22C	0.1870	1.1065	0.4838	0.026*	0.342 (12)
C23X	0.1700 (6)	0.9752 (17)	0.5524 (8)	0.029 (3)	0.342 (12)
H23C	0.2036	1.0011	0.5916	0.034*	0.342 (12)
C24X	0.1306 (3)	0.8728 (15)	0.5579 (4)	0.0333 (18)	0.342 (12)
H24C	0.1378	0.8349	0.6012	0.040*	0.342 (12)
C25X	0.0804 (3)	0.8241 (14)	0.5006 (3)	0.035 (2)	0.342 (12)
H25C	0.0552	0.7520	0.5044	0.042*	0.342 (12)
C26X	0.0705 (3)	0.8894 (11)	0.4379 (3)	0.0227 (14)	0.342 (12)
C27X	0.0197 (3)	0.8682 (12)	0.3690 (4)	0.0273 (16)	0.342 (12)
H1NA	0.5124 (11)	0.351 (3)	0.2695 (12)	0.025 (5)*	
H1OB	0.0450 (15)	0.749 (4)	0.1599 (18)	0.056 (9)*	
H1NB	−0.0342 (13)	1.066 (3)	0.2407 (14)	0.039 (7)*	
H1OA	0.4311 (13)	0.675 (3)	0.1226 (16)	0.045 (8)*	

### Atomic displacement parameters (Å^2^)

**Table:**

	*U*^11^	*U*^22^	*U*^33^	*U*^12^	*U*^13^	*U*^23^
F1A	0.0258 (5)	0.0597 (8)	0.0288 (5)	0.0058 (5)	0.0165 (4)	0.0077 (5)
F2A	0.0285 (6)	0.0497 (8)	0.0364 (6)	−0.0065 (5)	−0.0057 (5)	0.0214 (6)
O1A	0.0166 (5)	0.0276 (6)	0.0250 (5)	0.0069 (4)	0.0063 (4)	−0.0011 (4)
O2A	0.0227 (5)	0.0169 (5)	0.0397 (7)	0.0032 (4)	0.0190 (5)	0.0006 (5)
O3A	0.0209 (5)	0.0303 (6)	0.0285 (6)	−0.0039 (5)	0.0086 (4)	−0.0093 (5)
O4A	0.0297 (6)	0.0501 (9)	0.0396 (7)	−0.0206 (6)	0.0186 (6)	−0.0213 (7)
N1A	0.0193 (5)	0.0159 (5)	0.0262 (6)	0.0013 (4)	0.0126 (5)	−0.0009 (5)
N2A	0.0183 (5)	0.0200 (6)	0.0237 (6)	−0.0035 (4)	0.0109 (5)	−0.0040 (5)
C1A	0.0204 (6)	0.0252 (7)	0.0201 (6)	0.0039 (5)	0.0082 (5)	−0.0006 (5)
C2A	0.0200 (6)	0.0328 (8)	0.0239 (7)	0.0049 (6)	0.0096 (6)	−0.0002 (6)
C3A	0.0198 (6)	0.0364 (9)	0.0222 (7)	−0.0011 (6)	0.0098 (5)	−0.0009 (6)
C4A	0.0219 (7)	0.0303 (8)	0.0262 (7)	0.0007 (6)	0.0101 (6)	0.0050 (6)
C5A	0.0204 (6)	0.0226 (7)	0.0273 (7)	0.0022 (5)	0.0103 (6)	0.0030 (6)
C6A	0.0174 (6)	0.0196 (6)	0.0196 (6)	−0.0004 (5)	0.0064 (5)	−0.0029 (5)
C7A	0.0173 (6)	0.0168 (6)	0.0202 (6)	−0.0002 (5)	0.0067 (5)	−0.0034 (5)
C8A	0.0167 (6)	0.0195 (6)	0.0192 (6)	0.0018 (5)	0.0047 (5)	−0.0018 (5)
C9A	0.0142 (5)	0.0171 (6)	0.0222 (6)	0.0019 (5)	0.0050 (5)	−0.0028 (5)
C10A	0.0157 (5)	0.0151 (6)	0.0223 (6)	0.0001 (4)	0.0081 (5)	−0.0022 (5)
C11A	0.0151 (5)	0.0149 (6)	0.0220 (6)	0.0004 (4)	0.0069 (5)	−0.0010 (5)
C12A	0.0158 (5)	0.0179 (6)	0.0218 (6)	0.0014 (5)	0.0077 (5)	−0.0010 (5)
C13A	0.0151 (5)	0.0180 (6)	0.0231 (6)	−0.0002 (5)	0.0072 (5)	0.0032 (5)
C14A	0.0196 (6)	0.0235 (7)	0.0218 (7)	−0.0008 (5)	0.0092 (5)	0.0039 (5)
C15A	0.0230 (7)	0.0343 (9)	0.0218 (7)	−0.0068 (6)	0.0076 (6)	0.0059 (6)
C16A	0.0202 (7)	0.0359 (9)	0.0303 (8)	−0.0048 (6)	0.0015 (6)	0.0153 (7)
C17A	0.0199 (7)	0.0245 (8)	0.0441 (10)	0.0032 (6)	0.0064 (7)	0.0115 (7)
C18A	0.0190 (6)	0.0201 (7)	0.0350 (8)	0.0013 (5)	0.0094 (6)	0.0042 (6)
C19A	0.0162 (6)	0.0151 (6)	0.0252 (7)	−0.0017 (5)	0.0088 (5)	−0.0030 (5)
C20A	0.0164 (6)	0.0167 (6)	0.0248 (7)	0.0002 (5)	0.0089 (5)	−0.0003 (5)
C21A	0.0180 (6)	0.0190 (6)	0.0237 (7)	0.0007 (5)	0.0098 (5)	0.0008 (5)
C22A	0.0200 (7)	0.0314 (8)	0.0302 (8)	−0.0005 (6)	0.0123 (6)	−0.0010 (6)
C23A	0.0275 (8)	0.0342 (9)	0.0346 (9)	0.0036 (7)	0.0201 (7)	0.0032 (7)
C24A	0.0391 (9)	0.0312 (9)	0.0304 (8)	0.0017 (7)	0.0228 (8)	0.0001 (7)
C25A	0.0365 (9)	0.0321 (9)	0.0282 (8)	−0.0073 (7)	0.0187 (7)	−0.0083 (7)
C26A	0.0239 (7)	0.0223 (7)	0.0247 (7)	−0.0032 (5)	0.0126 (6)	−0.0029 (6)
C27A	0.0226 (7)	0.0252 (7)	0.0271 (7)	−0.0067 (6)	0.0125 (6)	−0.0067 (6)
F1B	0.0284 (5)	0.0549 (8)	0.0146 (4)	0.0039 (5)	0.0031 (4)	0.0014 (4)
F2B	0.0402 (7)	0.0562 (8)	0.0434 (7)	0.0062 (6)	0.0253 (6)	−0.0177 (6)
O1B	0.0192 (5)	0.0426 (7)	0.0199 (5)	0.0090 (5)	0.0064 (4)	0.0035 (5)
O2B	0.0266 (6)	0.0232 (6)	0.0206 (5)	0.0025 (4)	0.0038 (4)	0.0028 (4)
N1B	0.0320 (7)	0.0231 (6)	0.0139 (5)	0.0007 (5)	0.0042 (5)	0.0005 (5)
C1B	0.0167 (6)	0.0271 (7)	0.0197 (6)	0.0020 (5)	0.0083 (5)	0.0008 (5)
C2B	0.0173 (6)	0.0309 (8)	0.0207 (7)	0.0023 (6)	0.0052 (5)	−0.0005 (6)
C3B	0.0227 (7)	0.0315 (8)	0.0144 (6)	0.0039 (6)	0.0040 (5)	0.0004 (6)
C4B	0.0224 (7)	0.0413 (10)	0.0175 (6)	−0.0001 (6)	0.0085 (5)	0.0048 (6)
C5B	0.0181 (6)	0.0369 (9)	0.0172 (6)	−0.0024 (6)	0.0062 (5)	0.0034 (6)
C6B	0.0174 (6)	0.0234 (7)	0.0153 (6)	0.0023 (5)	0.0063 (5)	0.0016 (5)
C7B	0.0173 (6)	0.0239 (7)	0.0160 (6)	0.0000 (5)	0.0067 (5)	0.0017 (5)
C8B	0.0185 (6)	0.0313 (8)	0.0176 (6)	0.0021 (6)	0.0083 (5)	0.0015 (6)
C9B	0.0166 (6)	0.0258 (7)	0.0183 (6)	0.0010 (5)	0.0069 (5)	0.0019 (5)
C10B	0.0194 (6)	0.0184 (6)	0.0152 (6)	−0.0010 (5)	0.0061 (5)	0.0009 (5)
C11B	0.0207 (6)	0.0174 (6)	0.0172 (6)	0.0004 (5)	0.0081 (5)	0.0002 (5)
C12B	0.0182 (6)	0.0222 (7)	0.0173 (6)	0.0017 (5)	0.0066 (5)	0.0003 (5)
C13B	0.0216 (6)	0.0215 (7)	0.0176 (6)	0.0007 (5)	0.0078 (5)	−0.0032 (5)
C14B	0.0318 (8)	0.0251 (7)	0.0233 (7)	0.0023 (6)	0.0152 (6)	−0.0019 (6)
C15B	0.0342 (9)	0.0371 (9)	0.0256 (8)	0.0010 (7)	0.0168 (7)	−0.0050 (7)
C16B	0.0249 (7)	0.0402 (10)	0.0276 (8)	0.0019 (7)	0.0120 (6)	−0.0136 (7)
C17B	0.0236 (7)	0.0280 (8)	0.0296 (8)	0.0054 (6)	0.0057 (6)	−0.0087 (6)
C18B	0.0214 (6)	0.0227 (7)	0.0227 (7)	0.0015 (5)	0.0052 (5)	−0.0030 (6)
C19B	0.0224 (6)	0.0187 (6)	0.0166 (6)	−0.0033 (5)	0.0055 (5)	0.0023 (5)
O3B	0.0236 (14)	0.0203 (19)	0.0250 (11)	0.0081 (13)	0.0175 (10)	0.0056 (12)
O4B	0.0345 (14)	0.061 (3)	0.0298 (12)	−0.0243 (16)	0.0094 (10)	0.0012 (14)
N2B	0.0137 (13)	0.0123 (14)	0.0111 (9)	0.0031 (11)	0.0045 (9)	0.0000 (10)
C20B	0.022 (2)	0.019 (3)	0.0083 (13)	0.0040 (17)	0.0074 (12)	0.0013 (13)
C21B	0.0114 (16)	0.024 (3)	0.017 (2)	−0.0079 (15)	0.0058 (14)	−0.0025 (16)
C22B	0.025 (3)	0.027 (3)	0.019 (2)	0.004 (2)	0.0098 (19)	0.0037 (18)
C23B	0.020 (2)	0.030 (3)	0.0182 (16)	0.000 (2)	0.0001 (13)	0.0004 (17)
C24B	0.0369 (19)	0.052 (3)	0.0166 (12)	−0.0129 (19)	0.0060 (12)	0.0039 (16)
C25B	0.0365 (19)	0.066 (3)	0.0178 (12)	−0.027 (2)	0.0075 (11)	0.0057 (15)
C26B	0.0249 (16)	0.031 (2)	0.0163 (11)	−0.0068 (14)	0.0062 (10)	0.0033 (13)
C27B	0.0251 (15)	0.033 (2)	0.0170 (11)	−0.0101 (14)	0.0070 (10)	0.0006 (13)
O3X	0.036 (3)	0.016 (3)	0.049 (4)	0.014 (2)	0.029 (2)	0.010 (3)
O4X	0.034 (3)	0.057 (5)	0.029 (3)	−0.028 (3)	−0.005 (2)	0.015 (3)
N2X	0.023 (3)	0.018 (3)	0.018 (2)	0.012 (2)	0.009 (2)	−0.001 (2)
C20X	0.022 (5)	0.012 (5)	0.055 (8)	−0.005 (4)	0.017 (5)	−0.006 (5)
C21X	0.052 (7)	0.034 (7)	0.012 (4)	0.009 (5)	0.017 (4)	0.001 (3)
C22X	0.008 (4)	0.022 (6)	0.029 (5)	−0.002 (3)	0.001 (3)	−0.011 (4)
C23X	0.020 (3)	0.041 (8)	0.021 (3)	0.013 (4)	0.005 (2)	0.002 (4)
C24X	0.023 (2)	0.053 (5)	0.021 (3)	0.000 (3)	0.0058 (19)	0.008 (3)
C25X	0.022 (2)	0.057 (6)	0.024 (3)	−0.006 (3)	0.0072 (19)	0.013 (3)
C26X	0.016 (2)	0.029 (4)	0.020 (2)	−0.003 (2)	0.0045 (17)	0.006 (2)
C27X	0.022 (3)	0.033 (4)	0.022 (3)	−0.004 (3)	0.005 (2)	0.004 (3)

### Geometric parameters (Å, º)

**Table:**

F1A—C3A	1.3585 (19)	C1B—C2B	1.392 (2)
F2A—C16A	1.359 (2)	C1B—C6B	1.399 (2)
O1A—C9A	1.3577 (18)	C1B—H1BA	0.9300
O1A—H1OA	0.82 (3)	C2B—C3B	1.377 (2)
O2A—C19A	1.2449 (18)	C2B—H2BA	0.9300
O3A—C20A	1.200 (2)	C3B—C4B	1.378 (2)
O4A—C27A	1.200 (2)	C4B—C5B	1.391 (2)
N1A—C19A	1.346 (2)	C4B—H4BA	0.9300
N1A—N2A	1.3723 (18)	C5B—C6B	1.396 (2)
N1A—H1NA	0.80 (3)	C5B—H5BA	0.9300
N2A—C27A	1.406 (2)	C6B—C7B	1.480 (2)
N2A—C20A	1.4123 (19)	C7B—C8B	1.386 (2)
C1A—C2A	1.387 (2)	C7B—C12B	1.401 (2)
C1A—C6A	1.400 (2)	C8B—C9B	1.391 (2)
C1A—H1AA	0.9300	C8B—H8BA	0.9300
C2A—C3A	1.383 (3)	C9B—C10B	1.415 (2)
C2A—H2AA	0.9300	C10B—C11B	1.421 (2)
C3A—C4A	1.376 (2)	C10B—C19B	1.482 (2)
C4A—C5A	1.389 (2)	C11B—C12B	1.393 (2)
C4A—H4AA	0.9300	C11B—C13B	1.493 (2)
C5A—C6A	1.400 (2)	C12B—H12B	0.9300
C5A—H5AA	0.9300	C13B—C18B	1.398 (2)
C6A—C7A	1.480 (2)	C13B—C14B	1.400 (2)
C7A—C8A	1.391 (2)	C14B—C15B	1.393 (2)
C7A—C12A	1.402 (2)	C14B—H14B	0.9300
C8A—C9A	1.392 (2)	C15B—C16B	1.379 (3)
C8A—H8AA	0.9300	C15B—H15B	0.9300
C9A—C10A	1.413 (2)	C16B—C17B	1.377 (3)
C10A—C11A	1.421 (2)	C17B—C18B	1.394 (2)
C10A—C19A	1.484 (2)	C17B—H17B	0.9300
C11A—C12A	1.392 (2)	C18B—H18B	0.9300
C11A—C13A	1.485 (2)	O3B—C20B	1.214 (5)
C12A—H12A	0.9300	O4B—C27B	1.197 (4)
C13A—C14A	1.393 (2)	N2B—C27B	1.408 (5)
C13A—C18A	1.402 (2)	N2B—C20B	1.410 (6)
C14A—C15A	1.394 (2)	C20B—C21B	1.463 (6)
C14A—H14A	0.9300	C21B—C22B	1.369 (7)
C15A—C16A	1.373 (3)	C21B—C26B	1.401 (5)
C15A—H15A	0.9300	C22B—C23B	1.400 (8)
C16A—C17A	1.375 (3)	C22B—H22B	0.9300
C17A—C18A	1.390 (3)	C23B—C24B	1.370 (7)
C17A—H17A	0.9300	C23B—H23B	0.9300
C18A—H18A	0.9300	C24B—C25B	1.401 (5)
C20A—C21A	1.485 (2)	C24B—H24B	0.9300
C21A—C22A	1.384 (2)	C25B—C26B	1.381 (4)
C21A—C26A	1.390 (2)	C25B—H25B	0.9300
C22A—C23A	1.400 (3)	C26B—C27B	1.484 (5)
C22A—H22A	0.9300	O3X—C20X	1.202 (12)
C23A—C24A	1.386 (3)	O4X—C27X	1.190 (8)
C23A—H23A	0.9300	N2X—C27X	1.402 (11)
C24A—C25A	1.398 (3)	N2X—C20X	1.407 (12)
C24A—H24A	0.9300	C20X—C21X	1.480 (12)
C25A—C26A	1.382 (2)	C21X—C22X	1.389 (12)
C25A—H25A	0.9300	C21X—C26X	1.395 (12)
C26A—C27A	1.487 (2)	C22X—C23X	1.410 (13)
F1B—C3B	1.3587 (18)	C22X—H22C	0.9300
F2B—C16B	1.357 (2)	C23X—C24X	1.384 (12)
O1B—C9B	1.3558 (19)	C23X—H23C	0.9300
O1B—H1OB	0.88 (3)	C24X—C25X	1.400 (9)
O2B—C19B	1.245 (2)	C24X—H24C	0.9300
N1B—C19B	1.349 (2)	C25X—C26X	1.385 (8)
N1B—N2B	1.363 (5)	C25X—H25C	0.9300
N1B—N2X	1.432 (11)	C26X—C27X	1.496 (8)
N1B—H1NB	0.91 (3)		
			
C9A—O1A—H1OA	109 (2)	C2B—C3B—C4B	123.13 (14)
C19A—N1A—N2A	116.80 (13)	C3B—C4B—C5B	118.07 (15)
C19A—N1A—H1NA	125.1 (17)	C3B—C4B—H4BA	121.0
N2A—N1A—H1NA	118.0 (17)	C5B—C4B—H4BA	121.0
N1A—N2A—C27A	122.39 (13)	C4B—C5B—C6B	120.99 (14)
N1A—N2A—C20A	123.09 (13)	C4B—C5B—H5BA	119.5
C27A—N2A—C20A	112.85 (12)	C6B—C5B—H5BA	119.5
C2A—C1A—C6A	121.59 (15)	C5B—C6B—C1B	118.86 (14)
C2A—C1A—H1AA	119.2	C5B—C6B—C7B	120.18 (13)
C6A—C1A—H1AA	119.2	C1B—C6B—C7B	120.92 (13)
C3A—C2A—C1A	117.94 (15)	C8B—C7B—C12B	118.82 (14)
C3A—C2A—H2AA	121.0	C8B—C7B—C6B	119.80 (13)
C1A—C2A—H2AA	121.0	C12B—C7B—C6B	121.34 (13)
F1A—C3A—C4A	118.46 (16)	C7B—C8B—C9B	120.65 (14)
F1A—C3A—C2A	118.65 (15)	C7B—C8B—H8BA	119.7
C4A—C3A—C2A	122.89 (15)	C9B—C8B—H8BA	119.7
C3A—C4A—C5A	118.18 (16)	O1B—C9B—C8B	116.02 (14)
C3A—C4A—H4AA	120.9	O1B—C9B—C10B	123.05 (13)
C5A—C4A—H4AA	120.9	C8B—C9B—C10B	120.93 (14)
C4A—C5A—C6A	121.40 (15)	C9B—C10B—C11B	118.48 (13)
C4A—C5A—H5AA	119.3	C9B—C10B—C19B	115.99 (13)
C6A—C5A—H5AA	119.3	C11B—C10B—C19B	125.53 (13)
C1A—C6A—C5A	117.98 (14)	C12B—C11B—C10B	118.96 (13)
C1A—C6A—C7A	121.14 (14)	C12B—C11B—C13B	116.33 (13)
C5A—C6A—C7A	120.89 (14)	C10B—C11B—C13B	124.69 (13)
C8A—C7A—C12A	117.94 (14)	C11B—C12B—C7B	122.08 (14)
C8A—C7A—C6A	121.18 (14)	C11B—C12B—H12B	119.0
C12A—C7A—C6A	120.83 (13)	C7B—C12B—H12B	119.0
C7A—C8A—C9A	121.18 (14)	C18B—C13B—C14B	118.75 (15)
C7A—C8A—H8AA	119.4	C18B—C13B—C11B	119.71 (14)
C9A—C8A—H8AA	119.4	C14B—C13B—C11B	121.49 (14)
O1A—C9A—C8A	115.45 (14)	C15B—C14B—C13B	120.54 (17)
O1A—C9A—C10A	123.68 (14)	C15B—C14B—H14B	119.7
C8A—C9A—C10A	120.86 (13)	C13B—C14B—H14B	119.7
C9A—C10A—C11A	118.21 (13)	C16B—C15B—C14B	118.62 (18)
C9A—C10A—C19A	116.23 (13)	C16B—C15B—H15B	120.7
C11A—C10A—C19A	125.55 (14)	C14B—C15B—H15B	120.7
C12A—C11A—C10A	119.21 (14)	F2B—C16B—C17B	118.84 (18)
C12A—C11A—C13A	115.87 (13)	F2B—C16B—C15B	118.30 (18)
C10A—C11A—C13A	124.52 (13)	C17B—C16B—C15B	122.85 (16)
C11A—C12A—C7A	122.42 (13)	C16B—C17B—C18B	117.99 (17)
C11A—C12A—H12A	118.8	C16B—C17B—H17B	121.0
C7A—C12A—H12A	118.8	C18B—C17B—H17B	121.0
C14A—C13A—C18A	118.89 (15)	C17B—C18B—C13B	121.24 (17)
C14A—C13A—C11A	120.77 (14)	C17B—C18B—H18B	119.4
C18A—C13A—C11A	119.99 (14)	C13B—C18B—H18B	119.4
C13A—C14A—C15A	120.73 (16)	O2B—C19B—N1B	119.59 (14)
C13A—C14A—H14A	119.6	O2B—C19B—C10B	121.91 (14)
C15A—C14A—H14A	119.6	N1B—C19B—C10B	118.46 (14)
C16A—C15A—C14A	118.26 (18)	N1B—N2B—C27B	124.5 (3)
C16A—C15A—H15A	120.9	N1B—N2B—C20B	121.4 (4)
C14A—C15A—H15A	120.9	C27B—N2B—C20B	113.4 (4)
F2A—C16A—C15A	118.06 (19)	O3B—C20B—N2B	127.0 (5)
F2A—C16A—C17A	118.72 (18)	O3B—C20B—C21B	128.0 (6)
C15A—C16A—C17A	123.20 (16)	N2B—C20B—C21B	105.0 (4)
C16A—C17A—C18A	118.10 (17)	C22B—C21B—C26B	119.9 (6)
C16A—C17A—H17A	121.0	C22B—C21B—C20B	131.0 (5)
C18A—C17A—H17A	121.0	C26B—C21B—C20B	108.5 (4)
C17A—C18A—C13A	120.81 (17)	C21B—C22B—C23B	118.7 (7)
C17A—C18A—H18A	119.6	C21B—C22B—H22B	120.7
C13A—C18A—H18A	119.6	C23B—C22B—H22B	120.7
O2A—C19A—N1A	119.49 (14)	C24B—C23B—C22B	121.1 (6)
O2A—C19A—C10A	121.43 (14)	C24B—C23B—H23B	119.5
N1A—C19A—C10A	119.00 (13)	C22B—C23B—H23B	119.5
O3A—C20A—N2A	124.66 (14)	C23B—C24B—C25B	121.0 (4)
O3A—C20A—C21A	130.74 (14)	C23B—C24B—H24B	119.5
N2A—C20A—C21A	104.60 (13)	C25B—C24B—H24B	119.5
C22A—C21A—C26A	121.34 (15)	C26B—C25B—C24B	117.3 (3)
C22A—C21A—C20A	129.87 (15)	C26B—C25B—H25B	121.3
C26A—C21A—C20A	108.77 (13)	C24B—C25B—H25B	121.3
C21A—C22A—C23A	117.16 (16)	C25B—C26B—C21B	121.8 (4)
C21A—C22A—H22A	121.4	C25B—C26B—C27B	129.0 (3)
C23A—C22A—H22A	121.4	C21B—C26B—C27B	109.2 (3)
C24A—C23A—C22A	121.41 (16)	O4B—C27B—N2B	125.0 (4)
C24A—C23A—H23A	119.3	O4B—C27B—C26B	131.4 (3)
C22A—C23A—H23A	119.3	N2B—C27B—C26B	103.6 (3)
C23A—C24A—C25A	121.07 (17)	C27X—N2X—C20X	113.4 (9)
C23A—C24A—H24A	119.5	C27X—N2X—N1B	120.0 (8)
C25A—C24A—H24A	119.5	C20X—N2X—N1B	125.5 (9)
C26A—C25A—C24A	117.23 (17)	O3X—C20X—N2X	119.1 (12)
C26A—C25A—H25A	121.4	O3X—C20X—C21X	135.5 (12)
C24A—C25A—H25A	121.4	N2X—C20X—C21X	105.2 (9)
C25A—C26A—C21A	121.78 (15)	C22X—C21X—C26X	122.5 (10)
C25A—C26A—C27A	129.37 (16)	C22X—C21X—C20X	128.3 (11)
C21A—C26A—C27A	108.85 (14)	C26X—C21X—C20X	107.4 (9)
O4A—C27A—N2A	125.13 (15)	C21X—C22X—C23X	115.6 (12)
O4A—C27A—C26A	130.23 (16)	C21X—C22X—H22C	122.2
N2A—C27A—C26A	104.65 (13)	C23X—C22X—H22C	122.2
C9B—O1B—H1OB	109 (2)	C24X—C23X—C22X	121.3 (12)
C19B—N1B—N2B	123.2 (2)	C24X—C23X—H23C	119.4
C19B—N1B—N2X	109.5 (3)	C22X—C23X—H23C	119.4
N2B—N1B—N2X	13.9 (3)	C23X—C24X—C25X	122.4 (9)
C19B—N1B—H1NB	122.3 (17)	C23X—C24X—H24C	118.8
N2B—N1B—H1NB	114.5 (18)	C25X—C24X—H24C	118.8
N2X—N1B—H1NB	128.1 (18)	C26X—C25X—C24X	116.3 (6)
C2B—C1B—C6B	120.78 (14)	C26X—C25X—H25C	121.8
C2B—C1B—H1BA	119.6	C24X—C25X—H25C	121.8
C6B—C1B—H1BA	119.6	C25X—C26X—C21X	121.2 (7)
C3B—C2B—C1B	118.16 (14)	C25X—C26X—C27X	129.2 (6)
C3B—C2B—H2BA	120.9	C21X—C26X—C27X	109.4 (7)
C1B—C2B—H2BA	120.9	O4X—C27X—N2X	125.7 (7)
F1B—C3B—C2B	118.45 (15)	O4X—C27X—C26X	130.7 (6)
F1B—C3B—C4B	118.42 (15)	N2X—C27X—C26X	103.6 (6)
			
C19A—N1A—N2A—C27A	−76.28 (19)	O1B—C9B—C10B—C11B	−176.72 (15)
C19A—N1A—N2A—C20A	87.92 (18)	C8B—C9B—C10B—C11B	3.3 (2)
C6A—C1A—C2A—C3A	−0.8 (3)	O1B—C9B—C10B—C19B	3.9 (2)
C1A—C2A—C3A—F1A	179.92 (16)	C8B—C9B—C10B—C19B	−176.07 (15)
C1A—C2A—C3A—C4A	−0.1 (3)	C9B—C10B—C11B—C12B	−2.0 (2)
F1A—C3A—C4A—C5A	−178.67 (16)	C19B—C10B—C11B—C12B	177.40 (14)
C2A—C3A—C4A—C5A	1.3 (3)	C9B—C10B—C11B—C13B	176.17 (15)
C3A—C4A—C5A—C6A	−1.7 (3)	C19B—C10B—C11B—C13B	−4.5 (2)
C2A—C1A—C6A—C5A	0.4 (2)	C10B—C11B—C12B—C7B	−0.2 (2)
C2A—C1A—C6A—C7A	−179.18 (15)	C13B—C11B—C12B—C7B	−178.43 (15)
C4A—C5A—C6A—C1A	0.9 (2)	C8B—C7B—C12B—C11B	0.9 (2)
C4A—C5A—C6A—C7A	−179.52 (15)	C6B—C7B—C12B—C11B	178.66 (15)
C1A—C6A—C7A—C8A	−162.32 (15)	C12B—C11B—C13B—C18B	−52.7 (2)
C5A—C6A—C7A—C8A	18.1 (2)	C10B—C11B—C13B—C18B	129.13 (17)
C1A—C6A—C7A—C12A	20.4 (2)	C12B—C11B—C13B—C14B	124.57 (17)
C5A—C6A—C7A—C12A	−159.18 (15)	C10B—C11B—C13B—C14B	−53.6 (2)
C12A—C7A—C8A—C9A	1.3 (2)	C18B—C13B—C14B—C15B	−0.9 (3)
C6A—C7A—C8A—C9A	−176.05 (14)	C11B—C13B—C14B—C15B	−178.20 (16)
C7A—C8A—C9A—O1A	176.28 (14)	C13B—C14B—C15B—C16B	0.3 (3)
C7A—C8A—C9A—C10A	−4.6 (2)	C14B—C15B—C16B—F2B	179.41 (17)
O1A—C9A—C10A—C11A	−175.94 (14)	C14B—C15B—C16B—C17B	0.3 (3)
C8A—C9A—C10A—C11A	5.0 (2)	F2B—C16B—C17B—C18B	−179.42 (16)
O1A—C9A—C10A—C19A	2.9 (2)	C15B—C16B—C17B—C18B	−0.3 (3)
C8A—C9A—C10A—C19A	−176.11 (13)	C16B—C17B—C18B—C13B	−0.3 (2)
C9A—C10A—C11A—C12A	−2.3 (2)	C14B—C13B—C18B—C17B	0.9 (2)
C19A—C10A—C11A—C12A	178.97 (14)	C11B—C13B—C18B—C17B	178.25 (15)
C9A—C10A—C11A—C13A	170.18 (14)	N2B—N1B—C19B—O2B	−4.3 (3)
C19A—C10A—C11A—C13A	−8.6 (2)	N2X—N1B—C19B—O2B	−2.1 (5)
C10A—C11A—C12A—C7A	−1.0 (2)	N2B—N1B—C19B—C10B	177.9 (2)
C13A—C11A—C12A—C7A	−174.04 (14)	N2X—N1B—C19B—C10B	−179.9 (4)
C8A—C7A—C12A—C11A	1.5 (2)	C9B—C10B—C19B—O2B	−25.2 (2)
C6A—C7A—C12A—C11A	178.84 (14)	C11B—C10B—C19B—O2B	155.45 (16)
C12A—C11A—C13A—C14A	128.03 (15)	C9B—C10B—C19B—N1B	152.55 (15)
C10A—C11A—C13A—C14A	−44.6 (2)	C11B—C10B—C19B—N1B	−26.8 (2)
C12A—C11A—C13A—C18A	−45.1 (2)	C19B—N1B—N2B—C27B	−91.8 (4)
C10A—C11A—C13A—C18A	142.28 (15)	N2X—N1B—N2B—C27B	−100 (2)
C18A—C13A—C14A—C15A	0.8 (2)	C19B—N1B—N2B—C20B	77.4 (6)
C11A—C13A—C14A—C15A	−172.33 (14)	N2X—N1B—N2B—C20B	69 (2)
C13A—C14A—C15A—C16A	0.2 (2)	N1B—N2B—C20B—O3B	3.6 (11)
C14A—C15A—C16A—F2A	177.24 (15)	C27B—N2B—C20B—O3B	173.9 (7)
C14A—C15A—C16A—C17A	−1.1 (3)	N1B—N2B—C20B—C21B	−174.7 (5)
F2A—C16A—C17A—C18A	−177.35 (16)	C27B—N2B—C20B—C21B	−4.4 (8)
C15A—C16A—C17A—C18A	1.0 (3)	O3B—C20B—C21B—C22B	11.1 (18)
C16A—C17A—C18A—C13A	0.1 (3)	N2B—C20B—C21B—C22B	−170.6 (12)
C14A—C13A—C18A—C17A	−1.0 (2)	O3B—C20B—C21B—C26B	−178.0 (8)
C11A—C13A—C18A—C17A	172.26 (15)	N2B—C20B—C21B—C26B	0.2 (8)
N2A—N1A—C19A—O2A	−6.3 (2)	C26B—C21B—C22B—C23B	4.5 (18)
N2A—N1A—C19A—C10A	176.82 (13)	C20B—C21B—C22B—C23B	174.5 (10)
C9A—C10A—C19A—O2A	−32.5 (2)	C21B—C22B—C23B—C24B	−1.0 (18)
C11A—C10A—C19A—O2A	146.25 (16)	C22B—C23B—C24B—C25B	−1.4 (12)
C9A—C10A—C19A—N1A	144.29 (14)	C23B—C24B—C25B—C26B	0.1 (8)
C11A—C10A—C19A—N1A	−36.9 (2)	C24B—C25B—C26B—C21B	3.5 (8)
N1A—N2A—C20A—O3A	8.2 (2)	C24B—C25B—C26B—C27B	−178.5 (5)
C27A—N2A—C20A—O3A	173.73 (16)	C22B—C21B—C26B—C25B	−5.9 (12)
N1A—N2A—C20A—C21A	−171.17 (13)	C20B—C21B—C26B—C25B	−178.0 (6)
C27A—N2A—C20A—C21A	−5.62 (17)	C22B—C21B—C26B—C27B	175.7 (9)
O3A—C20A—C21A—C22A	3.3 (3)	C20B—C21B—C26B—C27B	3.7 (8)
N2A—C20A—C21A—C22A	−177.46 (17)	N1B—N2B—C27B—O4B	−5.8 (7)
O3A—C20A—C21A—C26A	−175.39 (18)	C20B—N2B—C27B—O4B	−175.8 (6)
N2A—C20A—C21A—C26A	3.90 (17)	N1B—N2B—C27B—C26B	176.4 (4)
C26A—C21A—C22A—C23A	0.6 (3)	C20B—N2B—C27B—C26B	6.4 (6)
C20A—C21A—C22A—C23A	−177.93 (17)	C25B—C26B—C27B—O4B	−1.7 (8)
C21A—C22A—C23A—C24A	0.0 (3)	C21B—C26B—C27B—O4B	176.4 (6)
C22A—C23A—C24A—C25A	−0.9 (3)	C25B—C26B—C27B—N2B	175.8 (5)
C23A—C24A—C25A—C26A	1.1 (3)	C21B—C26B—C27B—N2B	−6.0 (6)
C24A—C25A—C26A—C21A	−0.6 (3)	C19B—N1B—N2X—C27X	−90.1 (6)
C24A—C25A—C26A—C27A	178.80 (19)	N2B—N1B—N2X—C27X	82 (2)
C22A—C21A—C26A—C25A	−0.3 (3)	C19B—N1B—N2X—C20X	102.4 (11)
C20A—C21A—C26A—C25A	178.50 (16)	N2B—N1B—N2X—C20X	−85 (2)
C22A—C21A—C26A—C27A	−179.78 (16)	C27X—N2X—C20X—O3X	179.1 (13)
C20A—C21A—C26A—C27A	−0.99 (19)	N1B—N2X—C20X—O3X	−13 (2)
N1A—N2A—C27A—O4A	−9.3 (3)	C27X—N2X—C20X—C21X	−5.4 (16)
C20A—N2A—C27A—O4A	−174.96 (18)	N1B—N2X—C20X—C21X	162.9 (10)
N1A—N2A—C27A—C26A	170.70 (14)	O3X—C20X—C21X—C22X	−11 (4)
C20A—N2A—C27A—C26A	5.04 (18)	N2X—C20X—C21X—C22X	175 (2)
C25A—C26A—C27A—O4A	−1.8 (3)	O3X—C20X—C21X—C26X	−176 (2)
C21A—C26A—C27A—O4A	177.7 (2)	N2X—C20X—C21X—C26X	9.6 (18)
C25A—C26A—C27A—N2A	178.24 (18)	C26X—C21X—C22X—C23X	−9 (3)
C21A—C26A—C27A—N2A	−2.32 (19)	C20X—C21X—C22X—C23X	−171.6 (19)
C6B—C1B—C2B—C3B	−0.5 (3)	C21X—C22X—C23X—C24X	3 (3)
C1B—C2B—C3B—F1B	−179.48 (16)	C22X—C23X—C24X—C25X	3 (2)
C1B—C2B—C3B—C4B	0.9 (3)	C23X—C24X—C25X—C26X	−2.9 (14)
F1B—C3B—C4B—C5B	−179.94 (17)	C24X—C25X—C26X—C21X	−2.5 (15)
C2B—C3B—C4B—C5B	−0.4 (3)	C24X—C25X—C26X—C27X	−176.2 (8)
C3B—C4B—C5B—C6B	−0.7 (3)	C22X—C21X—C26X—C25X	9 (2)
C4B—C5B—C6B—C1B	1.1 (3)	C20X—C21X—C26X—C25X	174.8 (11)
C4B—C5B—C6B—C7B	179.01 (17)	C22X—C21X—C26X—C27X	−176.6 (17)
C2B—C1B—C6B—C5B	−0.5 (3)	C20X—C21X—C26X—C27X	−10.4 (16)
C2B—C1B—C6B—C7B	−178.39 (15)	C20X—N2X—C27X—O4X	179.8 (11)
C5B—C6B—C7B—C8B	−40.4 (2)	N1B—N2X—C27X—O4X	10.9 (12)
C1B—C6B—C7B—C8B	137.46 (17)	C20X—N2X—C27X—C26X	−0.8 (11)
C5B—C6B—C7B—C12B	141.92 (17)	N1B—N2X—C27X—C26X	−169.7 (5)
C1B—C6B—C7B—C12B	−40.2 (2)	C25X—C26X—C27X—O4X	0.7 (15)
C12B—C7B—C8B—C9B	0.4 (2)	C21X—C26X—C27X—O4X	−173.5 (12)
C6B—C7B—C8B—C9B	−177.31 (15)	C25X—C26X—C27X—N2X	−178.7 (8)
C7B—C8B—C9B—O1B	177.45 (16)	C21X—C26X—C27X—N2X	7.1 (11)
C7B—C8B—C9B—C10B	−2.6 (3)		

### Hydrogen-bond geometry (Å, º)

**Table:**

*D*—H···*A*	*D*—H	H···*A*	*D*···*A*	*D*—H···*A*
N1*A*—H1*NA*···O2*A*^i^	0.80 (3)	2.02 (3)	2.8000 (19)	166 (3)
O1*B*—H1*OB*···O2*B*	0.88 (3)	1.79 (4)	2.5663 (17)	145 (4)
N1*B*—H1*NB*···O2*B*^ii^	0.91 (3)	2.04 (3)	2.779 (2)	138 (2)
O1*A*—H1*OA*···O2*A*	0.82 (3)	1.94 (3)	2.6402 (18)	143 (3)
C2*A*—H2*AA*···F2*A*^iii^	0.93	2.45	3.160 (2)	133
C4*B*—H4*BA*···O3*B*^iv^	0.93	2.41	3.271 (7)	154
C14*B*—H14*B*···O3*B*^v^	0.93	2.44	3.293 (6)	152
C25*B*—H25*B*···O1*B*^vi^	0.93	2.47	3.209 (3)	136

Symmetry codes: (i) −*x*+1, *y*−1/2, −*z*+1/2; (ii) −*x*, *y*+1/2, −*z*+1/2; (iii) *x*, −*y*+1/2, *z*−1/2; (iv) −*x*, −*y*+2, −*z*; (v) −*x*, *y*−1/2, −*z*+1/2; (vi) *x*, −*y*+3/2, *z*+1/2.
